# Supplementary material for: O‐glycan initiation directs distinct biological pathways and controls epithelial differentiation
Source: EMBO Rep. 2020 Apr 23;21(6):e48885. doi: 10.15252/embr.201948885 (PMC7271655; doi:10.15252/embr.201948885)

Table of Contents

Legends	2
Appendix Table S1. Indel analysis and sequencing of *GALNT* KO cell lines	3
Appendix Figure S1. Overlap between different O-glycoproteomic approaches	5
Appendix Figure S2. Expression of *GALNT* genes (> 1 RPKM) in *GALNT* KO cell lines	6

Legends

Appendix Table S1. Indel analysis and sequencing of *GALNT* KO cell lines. Sequencing results for *GALNT* KO cell lines on WT or *COSMC* KO background. The table also contains information regarding the first predicted amino acid to be altered from indel formation. The clone suffixes A1-A3 refer to alleles 1-3, if heterozygotic indel formation is found.

Appendix Figure S1. Overlap between different O-glycoproteomic approaches. Venn diagrams depict overlap between proteins identified using the two O-glycoproteomic approaches for individual *GALNT* isoform KOs.

Appendix Figure S2. Expression of *GALNT* genes (> 1 RPKM) in *GALNT* KO cell lines. Bar graphs show average RPKM + SD.

Appendix Table S1. Indel analysis and sequencing of *GALNT* KO cell lines.

Gene	Clone	Sequence	gRNA/ZFN	Exon	Indel	First a.a.
GALNT1	REF	TTCCTGGATGCCCATTGTgagtgtACAGTGGGATGGCTGGAGCCTC	ZFN nuclease CTGGATGCCCATTGTgagtgtACAGT GGGATGGCTG	Total 11		559
	12C9.A1	TTCCTGGATGCCCA-----------CAGTGGGATGGCTGGAGCCTC		4	-11	211
	12C9.A2	TTCCTGG---------------------------------------		4	-129+12	208
	3E4.A1	-------------------------------------TGGAGCCTC		4	-108	185
	3E4.A2	--------------------------------ATGGCTGGAGCCTC		4	-104	184
	12G9.A1	TTCCTGGATGCCCA-----------CAGTGGGATGGCTGGAGCCTC		4	-11	211
	12G9.A2	TTCCTGG---------------------------------------		4	-129+12	208
	10G6	TTCCTGGATGCCCATTGT-ag-------------------------		4	-73	212
(In *COSMC* KO)	2E9.A1	----------------------------TGGGATGGCTGGAGCCTC		4	-42	202
	2E9.A2	TTCCTGGATGCCCA--------------------------------		4	-41	211

Gene	Clone	Sequence	gRNA/ZFN	Exon	Indel	First a.a.
GALNT2	REF	GGTCGGCCCTACTCAGGACcg--tggtCAGGTGAGGCCAGGAGATG	ZFN nuclease GTCGGCCCTACTCAGGACcgtggtCA GGTGAGGCCAGGAGAT	Total 16		571
	2A2	GGTCGGCCCT-ACTCAGGA-----------------------GATG		4	-22	155
	2D3.A1	GGTCGGCCCTACTCAGGACcgg-tggtCAGGTGAGGCCAGGAGATG		4	+1	156
	2D3.A2	GTCGG---		4	-53	151
	2D6.A1	GG---------------------------------------AGATG		4	-37	149
	2D6.A2	GGTCGGCC-T------------------------------------		4	-43	152
	10G5.A1	GGTCGGCCCTCACTCAG--------------GTGAGGCCAGGAGATG		4	-10	154
	10G5.A2	GGTCGGCCCT-ACTCT-------------------GGCCAGGAGATG		4	-16	152

(In *COSMC* KO)	1G8.A1	GGTCGGCCCT-ACTCAGGACcgTAtggtCAGGTGAGGCCAGGAGATG		4	+2	157
	1G8.A2	--------------------cg--tggtCAGGTGAGGCCAGGAGATG		4	-117	125
GALNT3	REF	TTTGAATTCAACAAACCTTCTcct----------------------	ZFN nuclease TTCAACAAACCTTCTccttatGGAAG TAACCATAAC	Total 11		633
	4.6B	TTTGAATTCAACAAACCTTCTcctCCGGACCCTAATTGACTGAGCG		5	+22	322
	REF	TTTGAATTCAACAAACCTTCTccttatGGAAGTAACCATAAC				
	6.7E	T------------------------atGGAAGTAACCATAAC		5	-24	314
	5.6D.A1	TTTGAATTCAACAAACCTTCTc---------------ATAAC		5	-15	321
	5.6D.A2	TTTGAATTCAACAAACCTTCTcctta--------ACCATAAC		5	-8	322(stop)
	5.6D.A3	TTTGAATTCAACAAACC-------------------------		5	-26	320
	9.8B.A1	TT-----------------------------GTAACCATAAC		5	-29	314
	9.8B.A2	TTTGAATTCAACAAACCTTCT---------------------		5	-33	321
	REF	TTTGAATTCAACAAACCTTCTcctt-atGGAAGTAACCATAA				
	2.9B	TTTGAATTCAACAAACCTTCTccttAatGGAAGTAACCATAA		5	+1	322(stop)
gRNA	REF	GAAAAGGTCTGATCACTGCTCGGTTGCTAG	gRNA1 CTGATCACTGCTCGGTTGCT			
	2.4D.A1	GAAAAGGTCTGATCACTGC-----TGCTAG		4	-5	261
	2.4D.A2	GAAAAGGTCTGATCACTGCTCGGTT(+131)GCTAG		4	+131	263
	2.4D.A3	GAAAAGGTCTGATCACTGCTCGGTG(+206)GCTAG		4	+206	262
	REF	TAGTTTTTCATAATGAA-GCGTGGT	gRNA2 TAGTTTTTCATAATGAAGCG			
	2.2H	TAGTTTTTCATAATGAAAGCGTGGT		3	+1	197
	REF	CTGTCCACAGTGTGCTCTATTCTTCACCTGCAATACTG--CTGAAGG	gRNA3 TTCACCTGCAATACTGCTGA			
	11H.A1	CTGTCCACAGTGTGCTCTATTCTTCACCTGCA------GTCTGAAGG		3	+2-6	215
	11H.A2	CT---		3	-84	205
(In *COSMC* KO)	REF	TTTGAATTCAACAAACCTTCTcct----------------------	ZFN nuclease TTCAACAAACCTTCTccttatGGAAG TAACCATAAC			
	3.3B	TTTGAATTCAACAAACCTTCTcctCCGGATCCTAATTGACTGAGCG		5	+22	322

Appendix Figure S1

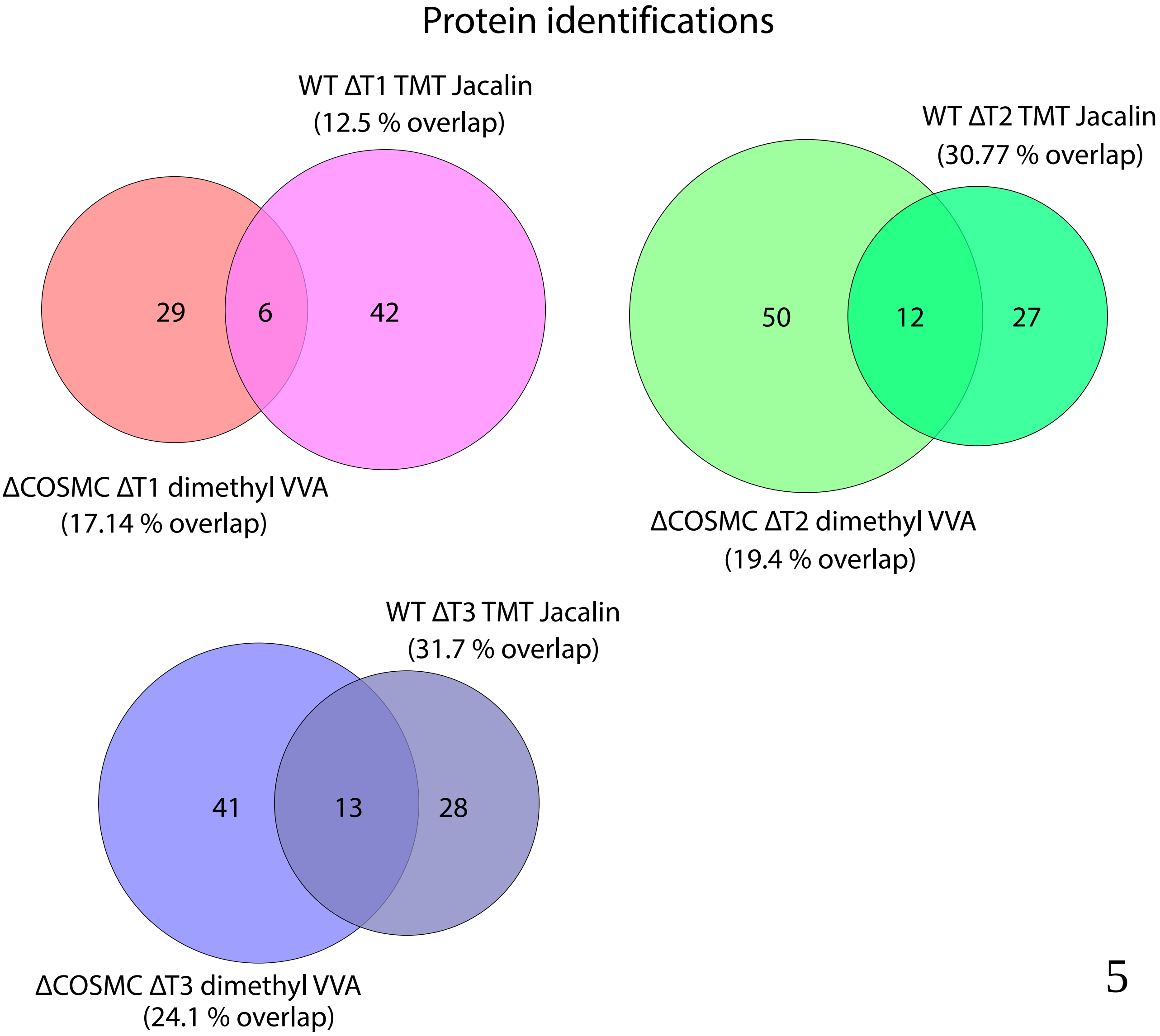

Appendix Figure S2

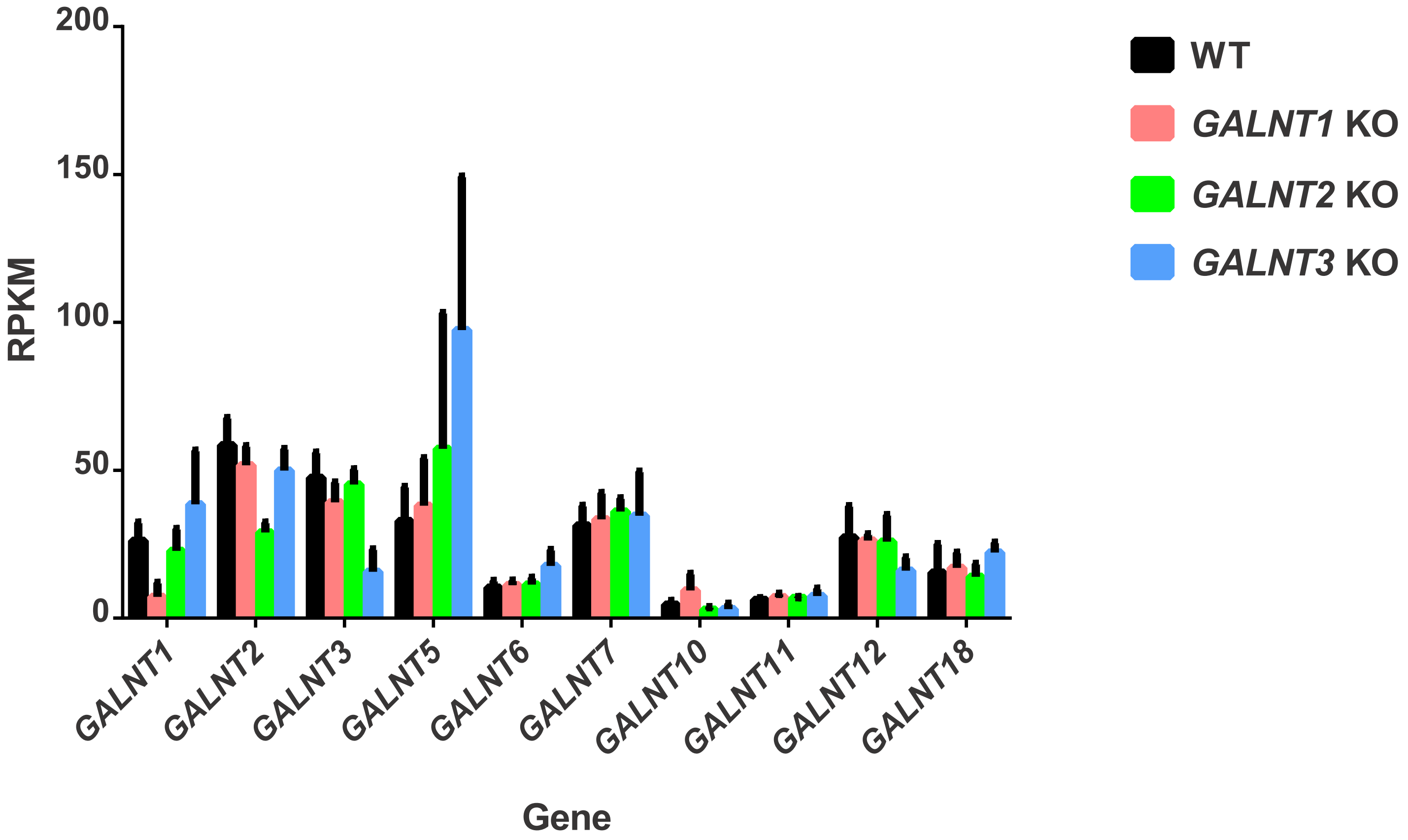

Supplement: Supplementary file 1 — Appendix [file EMBR-21-e48885-s001.pdf]
