# Supplementary material for: O‐glycan initiation directs distinct biological pathways and controls epithelial differentiation
Source: EMBO Rep. 2020 Apr 23;21(6):e48885. doi: 10.15252/embr.201948885 (PMC7271655; doi:10.15252/embr.201948885)
Supplement: Supplementary file 2 — Expanded View Figures PDF [file EMBR-21-e48885-s002.pdf]

Expanded View Figures

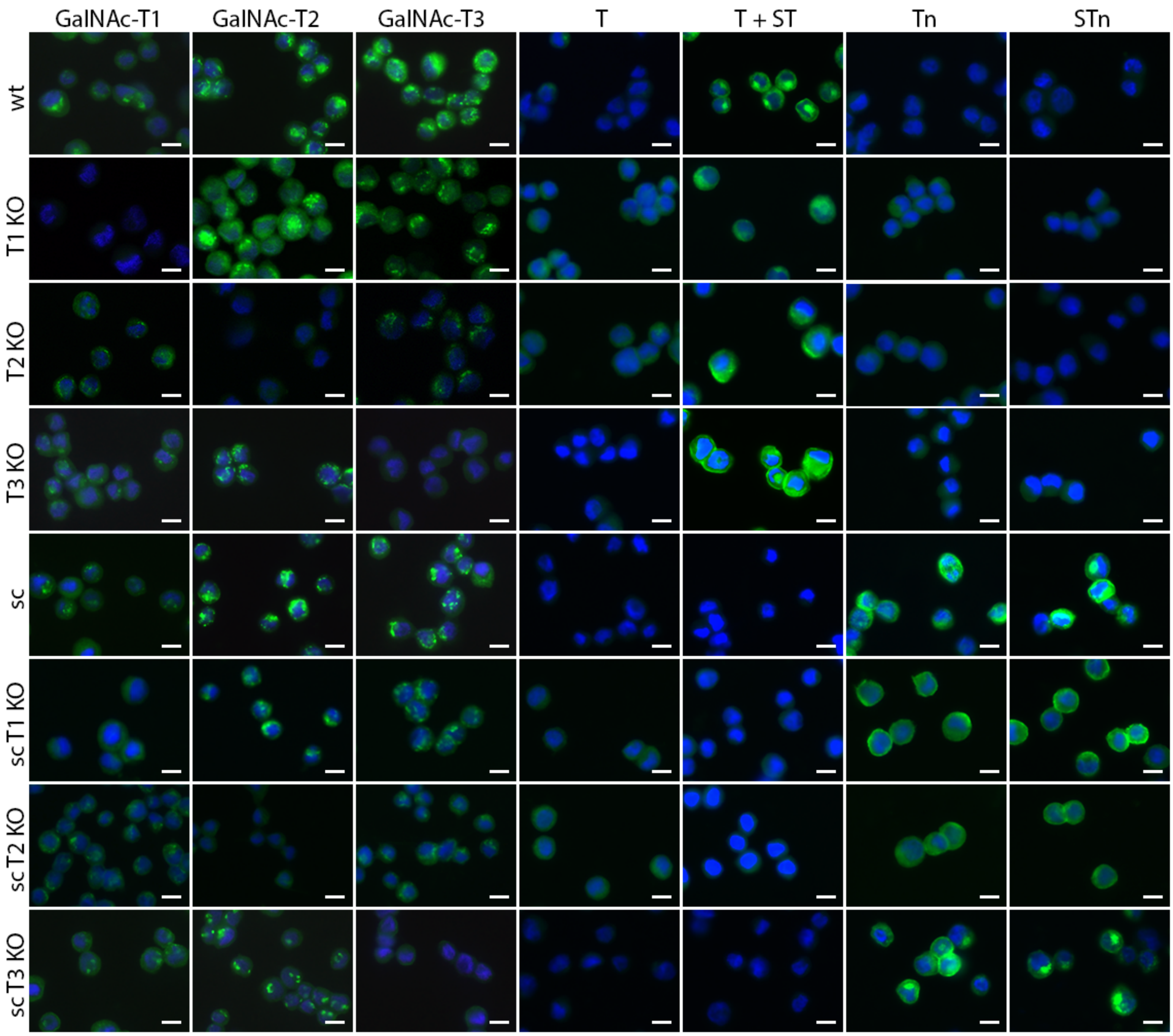

Figure EV1. Characterization of *GALNT* KO cell lines.

GALNT KO cell lines in WT or *COSMC* KO (sc, "SimpleCell") background were stained for GalNAc-T1, GalNAc-T2, and GalNAc-T3, as well as T (with (ST (sialyl-T) + T) or without (T) neuraminidase treatment), Tn, and STn (sialyl-Tn) glycoforms using monoclonal antibodies and lectins. Scale bar—10 μm.

 © 2020 The Authors

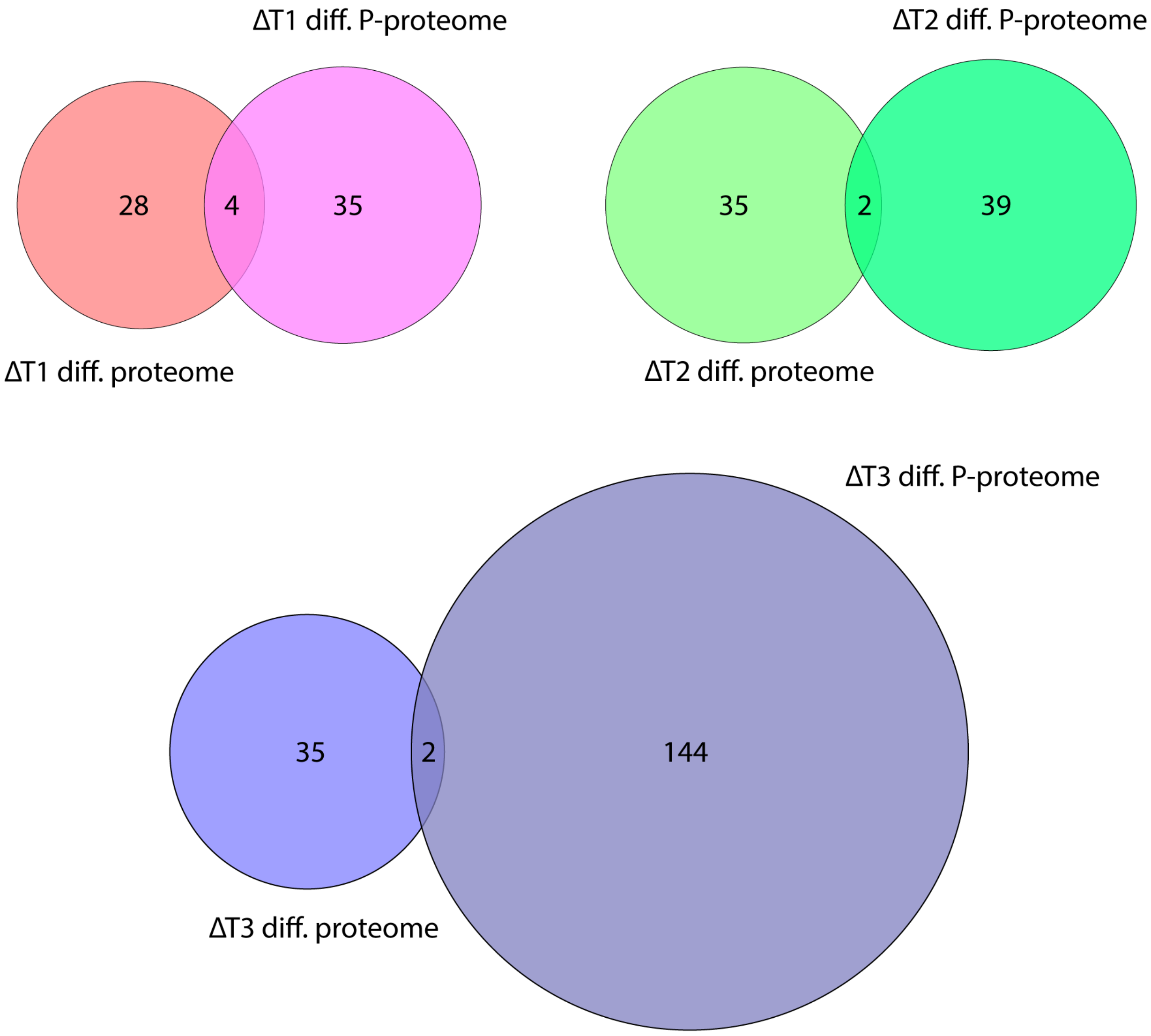

Figure EV2. Overlap between differential proteome and phosphoproteome.

Venn diagrams indicate overlap between proteins identified in differential proteomic and phosphoproteomic analyses of the individual *GALNT* isoform KOs.

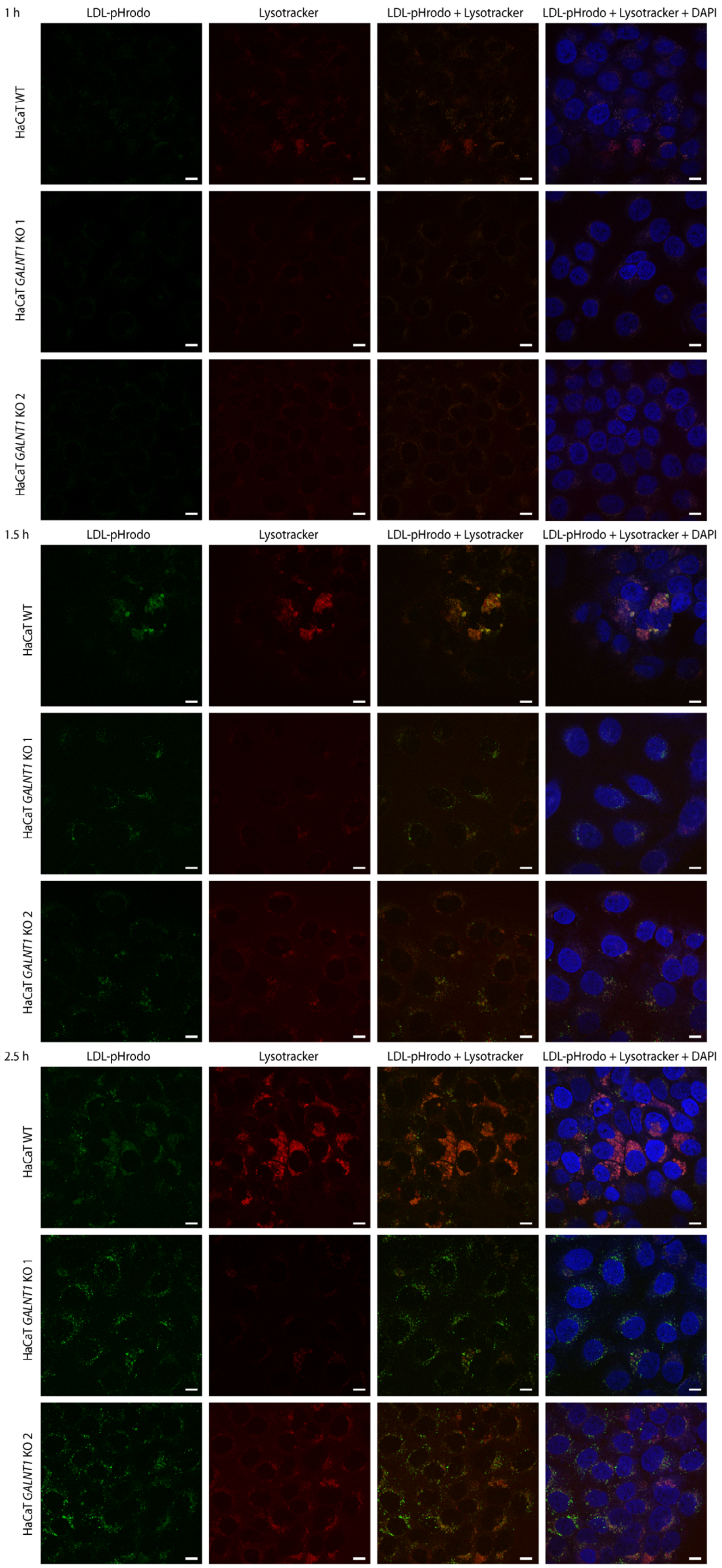

Figure EV3. LDL trafficking in HaCaT WT and *GALNT1* KO cells.

Cells grown on coverslips were cultured in media with lipoprotein-depleted serum for 24 h, followed by pulsing with Lysotracker Red DND-99 (−1 h) and LDL-pHrodo (0 h). Coverslips were fixed at 1 h (upper panels), 1.5 h (middle panels), and 2.5 h (lower panels) and imaged using confocal microscopy. Scale bar—10 μm.

 © 2020 The Authors

Figure EV4. Cell adhesion in HaCaT WT and *GALNT2* KO cells.

Cells grown on coverslips were fixed, permeabilized, and stained for cell–cell and cell–matrix adhesion molecules, followed by imaging by confocal microscopy. Green—relevant staining and blue—DAPI. Scale bar—50 μm.

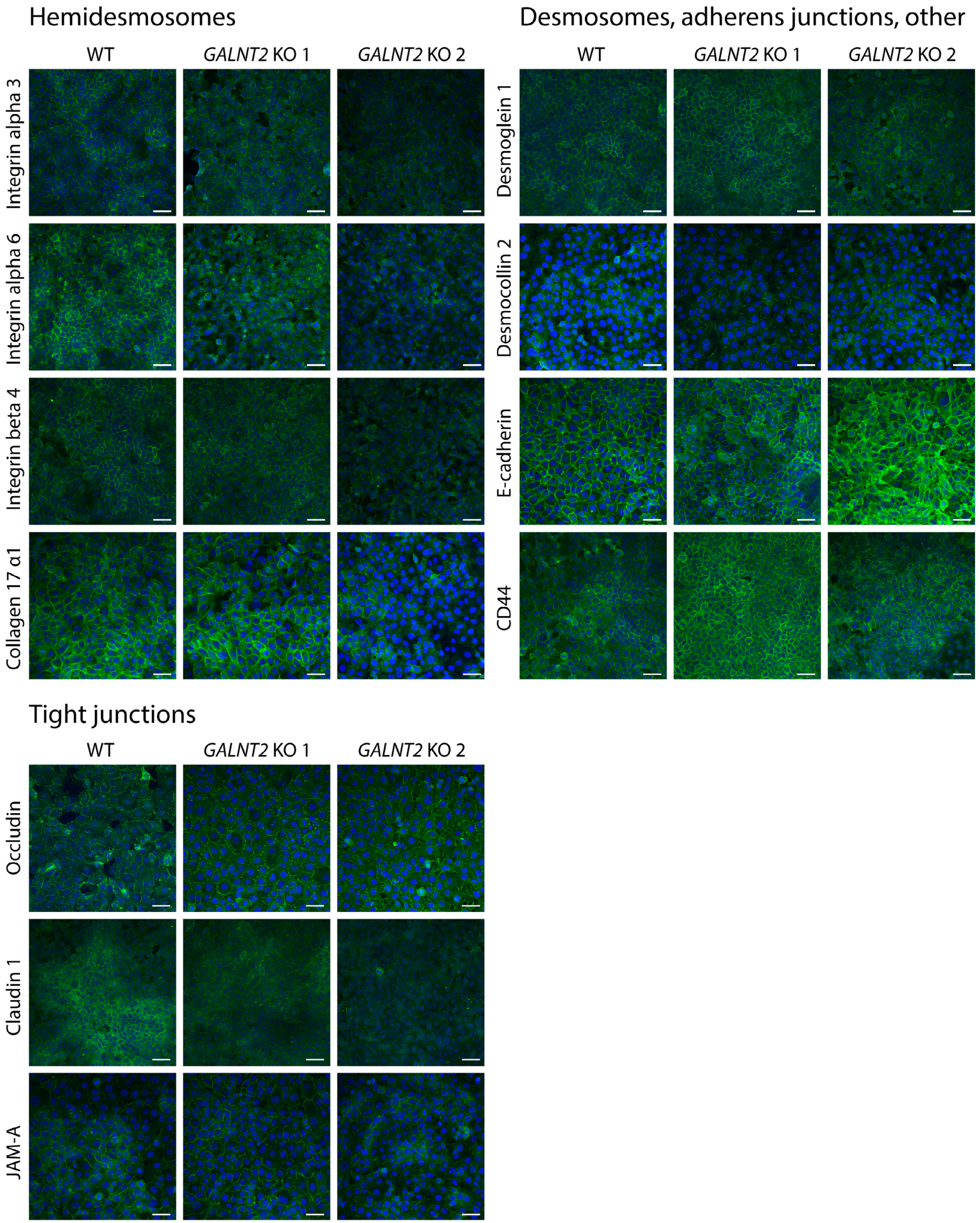

Figure EV4.

 © 2020 The Authors

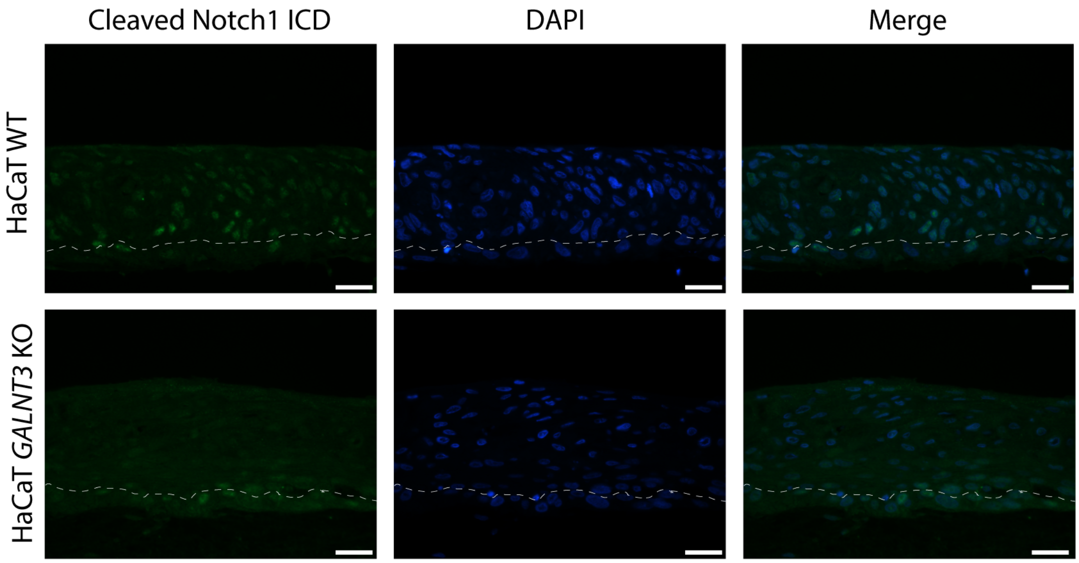

Figure EV5. Notch1 activity in HaCaT WT and *GALNT3* KO organotypic skin.

Immunofluorescence imaging of organotypic skin sections probed using cleaved Notch1 intracellular domain-specific mAb (green). Nuclei were counterstained with DAPI (blue). Scale bar—20 μm.
