# Supplementary material for: O‐glycan initiation directs distinct biological pathways and controls epithelial differentiation
Source: EMBO Rep. 2020 Apr 23;21(6):e48885. doi: 10.15252/embr.201948885 (PMC7271655; doi:10.15252/embr.201948885)
Supplement: Supplementary file 3 — Dataset EV1 [file EMBR-21-e48885-s003.zip › Dataset EV1.rtf]

Dataset EV1. RNA Seq. Gene expression data in RPKM is presented. In the “all genes normalized” tab, missing values were replaced with 0.001, and data analyzed using the CLC Genomics Workbench (Qiagen). In the individual KO tabs, statistically significant gene expression changes considering all three knock out clones of individual GALNTs (R package EdgeR analysis) are shown (filtered for at least 1 RPKM in at least one of the compared entries). logFC – log2(KO/WT).
