# Supplementary material for: O‐glycan initiation directs distinct biological pathways and controls epithelial differentiation
Source: EMBO Rep. 2020 Apr 23;21(6):e48885. doi: 10.15252/embr.201948885 (PMC7271655; doi:10.15252/embr.201948885)
Supplement: Supplementary file 4 — Dataset EV2 [file EMBR-21-e48885-s004.zip › Dataset EV2.rtf]

Dataset EV2. Differential phosphoproteomics. TMT 10-plex labeled TiO2-enriched phosphopeptides of two clones of each GALNT1 KO, GALNT2 KO, and GALNT3 KO were compared to wild type by tandem MS quantification. Data is presented as TMT ratios compared to “wild type 1” channel. Quantified individual phosphopeptide ratios outside 2x SD of individual clone variation consistently found in both clones and within 2x SD of the wild type variation were considered significant. Phosphoprotein identities also found changed in respective differential proteomic analyses were excluded from GO term enrichment analysis. GO terms with q value (Benjamini) of 0.05 and lower (0.1 and lower for GALNT1 KO) are shown.
