# Supplementary material for: O‐glycan initiation directs distinct biological pathways and controls epithelial differentiation
Source: EMBO Rep. 2020 Apr 23;21(6):e48885. doi: 10.15252/embr.201948885 (PMC7271655; doi:10.15252/embr.201948885)
Supplement: Supplementary file 5 — Dataset EV3 [file EMBR-21-e48885-s005.zip › Dataset EV3.rtf]

Dataset EV3. Differential proteomics. TMT 10-plex labeled peptides (before TiO2 enrichment) of two clones of each GALNT1 KO, GALNT2 KO, and GALNT3 KO were compared to wild type by tandem MS quantification. Proteins identified by at least two peptides were included in the analysis. Data is presented as average TMT ratios (based on quantification of corresponding peptides) for individual proteins compared to “wild type 1” channel. Quantified average ratios outside 2x SD of individual clone variation consistently found in both clones and within 2x SD of the wild type variation were considered significant.
