# Supplementary material for: O‐glycan initiation directs distinct biological pathways and controls epithelial differentiation
Source: EMBO Rep. 2020 Apr 23;21(6):e48885. doi: 10.15252/embr.201948885 (PMC7271655; doi:10.15252/embr.201948885)
Supplement: Supplementary file 6 — Dataset EV4 [file EMBR-21-e48885-s006.zip › Dataset EV4.rtf]

Dataset EV4. Differential glycoproteomics. TMT 10-plex labeled Jacalin-enriched glycopeptides of three clones of each GALNT1 KO, GALNT2 KO, and GALNT3 KO were compared to wild type by tandem MS quantification. Alternatively, dimethyl labeled VVA-enriched glycopeptides derived from isogenic cell line pairs in the COSMC KO background were compared. For the TMT dataset, quantified glycopeptide ratios lower than or equal to 0.5 in all three clones were considered significant. For the three dimethyl datasets, quantified glycopeptide ratios of log10(GALNT KO COSMC KO/COSMC KO) ≤-0.5 were considered significant. Identities of differentially glycosylated proteins identified in both methods were used for GO term enrichment analysis. Proteome quantification before Jacalin lectin weak affinity enrichment is included. Data is presented as average TMT ratios (based on quantification of corresponding peptides) for individual proteins compared to WT channel. 
